# Design and Biological Evaluation of a Gelatin/Recombinant Type III Collagen/CMC Composite Hydrogel for Wound Healing

**DOI:** 10.3390/gels12020142

**Published:** 2026-02-03

**Authors:** Ruixue Wu, Yunjie Shi, Yusi Hu, Jielei Han, Zhenyu Wang, Zhouguang Wang, Qian Xu

**Affiliations:** 1 School of Pharmaceutical Sciences, Wenzhou Medical University, Wenzhou 325035, China; 319197494@wmu.edu.cn (R.W.);; 2 Alberta Institute, Wenzhou Medical University, Wenzhou 325035, China

**Keywords:** composite hydrogel, gelatin, recombinant type III collagen, carboxymethyl cellulose, wound healing

## Abstract

Effective chronic skin wound healing remains challenging due to excessive inflammation, insufficient vascular support, and impaired extracellular matrix remodeling. By rationally designing and integrating complementary biomaterials, it is possible to synergistically tailor physicochemical properties and biological performance for tissue repair and regeneration. In this study, a gelatin-based composite hydrogel incorporating recombinant type III collagen (rColIII) and carboxymethyl cellulose (CMC) was developed via EDC/NHS-mediated crosslinking and evaluated for wound repair. By tuning the rColIII/CMC ratio, the hydrogel mechanical modulus (G′) increased from ~1.2 kPa to ~2.6 kPa, and enzymatic degradation could be modulated, as reflected by changes in the remaining material mass. The optimized Gel/rCol/CMC-1 formulation supported 3T3 cell migration (1.8-fold increase at 24 h) and promoted a pro-regenerative (M2-like) macrophage phenotype in vitro. In a full-thickness diabetic wound model, Gel/rCol/CMC-1 accelerated wound closure (82.3 ± 4.7% vs. 56.9 ± 5.1% at day 14) and enhanced tissue quality, evidenced by more organized collagen deposition and increased CD31^+^/α-SMA^+^ vessel density. These results demonstrate that formulation-driven tuning of gelatin/rColIII/CMC matrices creates a supportive microenvironment for coordinated wound repair, highlighting their potential as regenerative hydrogel dressings for difficult-to-heal wounds.

## 1. Introduction

The effective management of hard-to-treat skin wounds, including diabetic foot ulcers, venous leg ulcers, and pressure ulcers, remains a persistent challenge in clinical practice [1]. These wounds are commonly associated with prolonged healing times, frequent recurrence, and an increased risk of infection, leading to substantial physical, psychological, and economic burdens for both patients and healthcare systems worldwide [2,3]. Unlike acute wounds, hard-to-treat wounds are characterized by dysregulated inflammatory responses, insufficient vascular support, and impaired extracellular matrix (ECM) remodeling, which together disrupt the normal progression of tissue repair [4,5]. Despite continuous advances in wound care strategies, there remains a significant unmet clinical need for safe and effective therapeutic approaches capable of supporting coordinated and durable wound healing [6].

Wound healing is a dynamic and highly regulated biological process that involves overlapping and interdependent phases of inflammation, proliferation, and tissue remodeling [4]. Throughout this process, the local wound microenvironment plays a critical role in governing cell migration, angiogenesis, immune regulation, and extracellular matrix deposition. In chronic or hard-to-treat wounds, disruption of this microenvironment often results in prolonged inflammation and ineffective tissue repair [7]. Conventional wound dressings are primarily designed to act as passive physical barriers, providing protection against external contamination and moisture loss. However, such dressings generally lack the capacity to actively regulate cellular behavior or modulate the wound microenvironment [8]. In recent years, biomaterial-based wound dressings and scaffolds have attracted increasing attention because they can offer temporary structural support while simultaneously interacting with cells and tissues [9]. Nevertheless, the rational design of biomaterials that integrate mechanical stability, controlled degradation, and biologically relevant functionality remains a major challenge in the development of advanced wound healing strategies.

An ideal wound dressing or scaffold should possess sufficient mechanical integrity to maintain structural stability under physiological conditions, while degrading at a rate compatible with tissue regeneration [10,11]. Materials that degrade too rapidly may fail to provide sustained support for cell infiltration and extracellular matrix deposition, whereas materials that persist excessively may impede tissue remodeling and prolong inflammatory responses. Beyond mechanical strength and degradation behavior, wound dressings are also expected to create a favorable microenvironment that supports cell adhesion, migration, and proliferation, promotes angiogenesis, and modulates immune responses toward a regenerative phenotype [12]. Balancing these often competing requirements within a single material system remains particularly challenging, reflecting the complexity and dynamic nature of the wound healing process.

Natural biomaterials derived from extracellular matrix (ECM) components have been extensively investigated for wound healing applications owing to their inherent biocompatibility and biological relevance [13,14]. Among them, gelatin, a denatured form of collagen, is one of the most widely used protein-based biomaterials in tissue engineering and regenerative medicine [15]. Gelatin is abundant, cost-effective, and readily processable into hydrogels with tunable mechanical properties and degradation profiles, making it attractive for wound dressing and scaffold fabrication. Moreover, gelatin retains cell-interactive motifs that facilitate cell adhesion and migration, which are essential for tissue repair [16,17]. Importantly, gelatin-based materials have already been translated into commercially available products, including wound dressings and hemostatic agents, demonstrating their practical applicability and clinical acceptance. However, gelatin-based hydrogels alone often exhibit limited mechanical robustness and lack sufficient biological specificity to support the coordinated regulation of inflammation, angiogenesis, and matrix remodeling, particularly in complex or hard-to-treat wounds. These limitations highlight the need to further modify or combine gelatin with complementary components to enhance its functional performance.

Recombinant collagen has emerged as an attractive alternative to animal-derived collagen in biomedical applications, owing to its defined molecular composition, reduced batch-to-batch variability, and elimination of risks associated with animal-derived pathogens and immunogenic contaminants [18]. Compared with collagen extracted from animal tissues, recombinant collagen offers improved reproducibility and quality control, which are particularly important for translational and clinical applications [19]. Among different collagen subtypes, recombinant type III collagen (rColIII) is of special relevance to wound healing, as type III collagen is a predominant component of the provisional extracellular matrix formed during the early stages of tissue repair. Type III collagen plays a critical role in supporting cell migration, granulation tissue formation, and subsequent matrix remodeling during wound healing [20,21]. Its presence is closely associated with early regenerative responses and the transition toward more mature collagen architectures at later stages. rColIII scaffolds have been shown to promote fibroblast adhesion, proliferation, and migration while maintaining favorable biocompatibility and low immunogenicity, making them promising biomaterials for soft regenerative applications [22,23,24]. Hybrid hydrogels incorporating rColIII have been shown to accelerate wound closure, enhance collagen deposition, and improve vascularization in full-thickness diabetic wound models without eliciting adverse systemic reactions, underscoring their in vivo efficacy in challenging healing environments [25,26]. In addition, rColIII has been reported to facilitate functional skin regeneration in both preclinical and clinical studies, exhibiting enhanced re-epithelialization, reduced inflammation, and improved dermal matrix organization in models of skin injury and UV-induced damage [24,27]. Despite these advantages, recombinant collagen-based matrices alone often provide limited control over the physicochemical microenvironment, highlighting the need for complementary components to further tailor material properties for wound healing.

Beyond protein-based components, polysaccharide materials are frequently introduced into wound dressings to modulate hydration, microstructure, and the local wound microenvironment [28]. Such materials can absorb excess wound exudate, maintain a moist interface, and influence the viscoelastic behavior of hydrogel matrices, thereby supporting cell–material interactions during tissue repair. When integrated into composite hydrogels, polysaccharides may serve as effective modulators of network organization and physicochemical properties rather than primary sources of biological activity [29,30]. Among these materials, carboxymethyl cellulose (CMC) has been widely applied in tissue engineering field owing to its hydrophilicity, fluid absorption capacity, and established clinical use [31,32]. In composite systems, the contribution of CMC is highly dependent on its incorporation level and interaction with protein-based matrices [29]. Excessive polysaccharide content may disrupt hydrogel network integrity and adversely affect mechanical performance or cellular responses, underscoring the importance of rational formulation design when integrating CMC into multifunctional wound matrices.

From a material design perspective, the integration of protein-based matrices with polysaccharide components provides a rational strategy to address the multifaceted requirements of wound healing materials. By combining gelatin as a structural scaffold, rColIII as a biologically relevant extracellular matrix component, and polysaccharide-based modulators of hydration and network organization, composite hydrogels offer the potential to achieve balanced physicochemical properties and coordinated biological responses. However, systematic investigations that correlate formulation design with material performance and biological outcomes in wound healing remain limited. Recent studies have demonstrated that rCol III-based hydrogels can enhance dermal regeneration through improved ECM mimicry and reduced inflammation, and that polysaccharide-based hydrogels, such as CMC, can provide tunable mechanics, hydration, and degradation [25,26,33]. However, these systems are generally evaluated at isolated material or biological levels. Systematic integration of ECM components with tunable polysaccharide content, together with multi-scale evaluation linking formulation-dependent physicochemical properties to cellular responses and in vivo wound repair, remains largely unexplored.

**Scheme 1:**
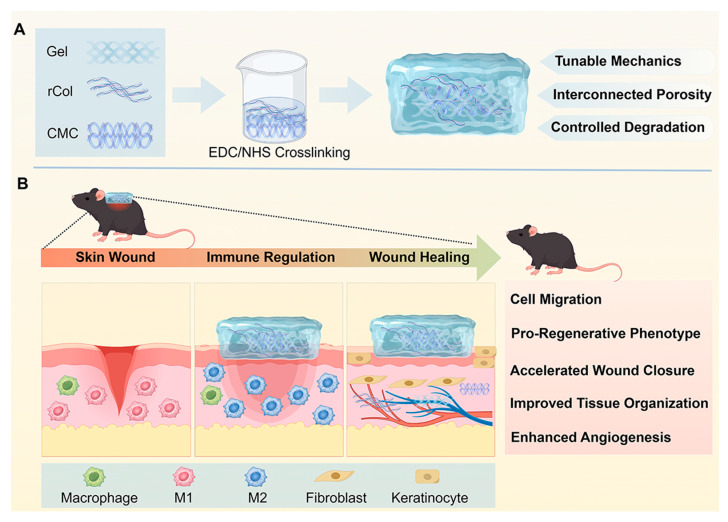
Schematic illustration of the design and regenerative function of the composite hydrogel. (**A**) Formation of a composite hydrogel. (**B**) Application of the composite hydrogel to diabetic wounds in mice.

In this study, we developed a series of gelatin-based composite hydrogels incorporating rColIII and CMC via EDC/NHS-mediated crosslinking, aiming to integrate complementary structural and microenvironmental functions for wound healing, as schematically illustrated in Scheme 1. By systematically varying the composition of the hydrogel network, we evaluated the resulting changes in mechanical properties, microstructure, cytocompatibility, and cellular responses in vitro, as well as wound healing performance in a full-thickness skin wound model in diabetic mice. Through this formulation-driven evaluation, the present work aims to provide insight into the rational design of composite hydrogels as regenerative wound matrices for hard-to-treat wounds.

## 2. Results and Discussion

### 2.1. Fabrication and Mechanical Characterization of Gel/rCol/CMC Composite Hydrogels

**Figure 1:**
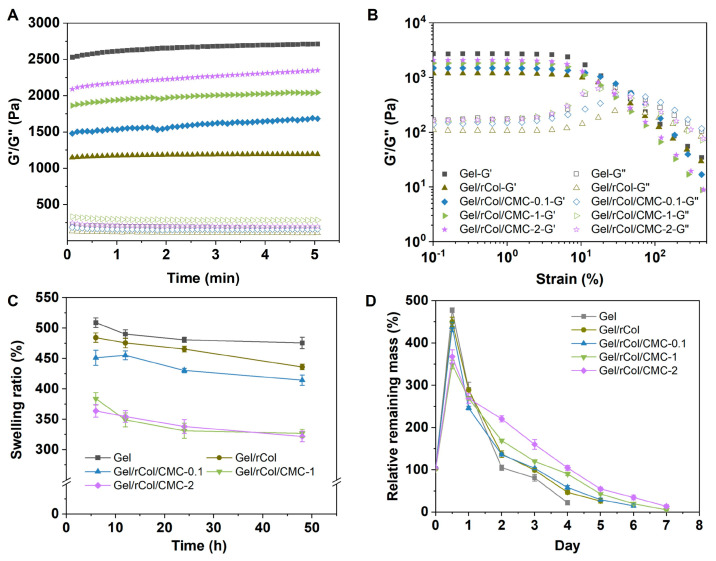
Rheological behavior, swelling capacity, and degradation profiles of different composite hydrogels. (**A**) Time-dependent evaluation of storage modulus (G′) and loss modulus (G″) over 5 min. (**B**) Strain-dependent G′ and G″ measured by amplitude sweep, showing the linear viscoelastic region and yielding behavior of the hydrogels. (**C**) Swelling ratio of hydrogels incubated in PBS medium over time. (**D**) Relative remaining mass (%) of hydrogels during in vitro degradation. Gelatin (Gel), gelatin/rColIII (Gel/rCol), and Gel/rCol/CMC hydrogels with different CMC contents (0.1, 1, and 2).

Gelatin-based composite hydrogels incorporating recombinant type III collagen (rColIII) and carboxymethyl cellulose (CMC) were successfully prepared via EDC/NHS-mediated crosslinking (Figure S1). Scaffolds formed by gelatin alone and gelatin with rCol were also fabricated as the control groups. The EDC/NHS method is a commonly used approach for crosslinking collagen or gelatin molecules with carboxyl and amino groups to form stable amide bonds. It is a highly efficient reaction and offers many advantages, including minimal toxicity with no harmless byproducts, highly controllability, mild reaction conditions, and preservation of the natural structure and biological activity. The viscoelastic properties of the resulting hydrogels were first evaluated to assess the formation and stability of the polymer networks (Figure 1A,B). All formulations exhibited typical solid-like behavior, with the storage modulus (G′) consistently exceeding the loss modulus (G″) across the measurements, indicating successful crosslinking and stable hydrogel network formation. The Gel hydrogel (gelatin alone) showed a relatively high initial G′, which can be attributed to the dense physical gelation and efficient covalent crosslinking of gelatin chains. Upon further incorporation of CMC, the mechanical properties of the hydrogels became tunable rather than simply enhanced or weakened. Among the composite hydrogels, Gel/rCol/CMC-2 showed a higher G′ than Gel/rCol/CMC-1 and Gel/rCol/CMC-0. The increase in G′ with increasing CMC content reflects reinforcement of the primary gelatin network through polysaccharide-mediated chain entanglement and non-covalent interactions. At higher CMC concentrations, the gelatin–CMC hybrid network became denser and more tightly packed, contributing to enhanced mechanical rigidity in a composition-dependent manner. Strain sweep measurements revealed that all hydrogels maintained elastic-dominant behavior within a defined linear viscoelastic region, followed by gradual yielding at higher strain. Collectively, these results demonstrate that the viscoelastic properties of the hydrogels can be effectively regulated by compositional design while preserving mechanical stability under deformation.

In addition to mechanical behavior, the swelling characteristics of the hydrogels were examined to evaluate their hydration capacity (Figure 1C). All hydrogels exhibited rapid initial swelling, followed by gradual stabilization, reflecting the hydrophilic nature of the polymer networks. Incorporation of CMC altered the swelling ratio in a composition-dependent manner, with higher CMC content leading to reduced swelling capacity. This observation indicates that polysaccharide incorporation modulates hydrogel–water interactions and network expansion, rather than simply increasing water uptake. The in vitro degradation profiles further demonstrated formulation-dependent differences in structural stability (Figure 1D). While all hydrogels underwent progressive mass loss over time, Gel/rCol/CMC hydrogels displayed slower degradation compared with Gel and Gel/rCol formulations. Notably, moderate CMC incorporation contributed to improved structural retention, whereas excessive CMC did not proportionally enhance stability, again highlighting the importance of formulation balance. From a practical perspective, the ability to fine-tune mechanical properties, hydration behavior, and degradation kinetics without compromising structural integrity is critical for wound dressing materials, which must remain stable while accommodating tissue movement and dynamic wound environments.

### 2.2. Microstructural Characteristics of Composite Hydrogels

**Figure 2:**
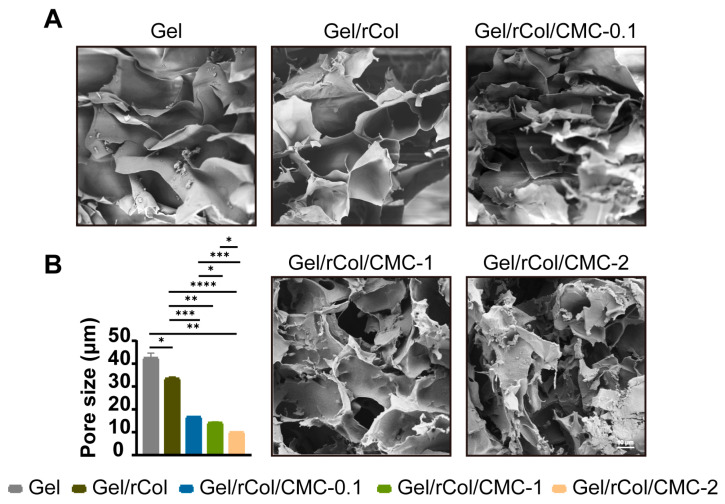
Microstructural characterization of Gel/rCol/CMC composite hydrogels. (**A**) Representative scanning electron microscopy (SEM) images of gelatin (Gel), gelatin/recombinant type III collagen (Gel/rCol), and Gel/rCol/CMC hydrogels with different CMC contents (0.1, 1, and 2), showing the internal porous architecture of freeze-dried hydrogels. (**B**) Quantitative analysis of pore size distribution based on SEM images. Scale bar: 10 μm. Data are presented as mean ± SD, and statistical significance is indicated (* *p* < 0.05, ** *p* < 0.01, *** *p* < 0.001, **** *p* < 0.0001).

The internal microstructures of the Gel/rCol/CMC hydrogels were examined by scanning electron microscopy (SEM) to further elucidate the influence of rColIII and CMC incorporation on network architecture (Figure 2). The Gel hydrogel exhibited a relatively loose and heterogeneous porous structure, characterized by large and irregularly distributed pores. Such a morphology is consistent with the physical gelation behavior of gelatin and reflects a lower level of structural organization within the network. In contrast, the introduction of rColIII and CMC led to noticeable changes in pore morphology and distribution. Composite hydrogels displayed a more compact and interconnected porous network, with reduced pore size and improved uniformity. Among the tested formulations, Gel/rCol/CMC-1 showed a well-balanced microstructure. This architectural feature suggests enhanced intermolecular interactions and crosslinking density within the composite matrix, without causing excessive densification. A more uniform and interconnected porous architecture is generally favorable for maintaining mechanical stability while allowing sufficient space for nutrient diffusion and cell infiltration. This provides a structural basis for the subsequent biological performance of Gel/rCol/CMC-1 observed in later in vitro and in vivo evaluations.

From a biological perspective, pore size and structural uniformity are critical parameters influencing cell infiltration, nutrient transport, and tissue integration. The observed differences in microarchitecture among Gel/rCol/CMC hydrogels provide a structural basis for understanding the formulation-dependent cellular responses and wound healing outcomes reported in subsequent sections. Together, these results indicate that rational modulation of CMC content enables fine control over hydrogel microstructure, rather than simply increasing scaffold density or compactness. Based on these abovementioned physicochemical characteristics, Gel/rCol/CMC-0.1 and Gel/rCol/CMC-1 were selected as a representative formulation for subsequent in vitro and in vivo evaluations related to wound healing.

### 2.3. In Vitro Cell Survival and Proliferation of the Composite Hydrogels

**Figure 3:**
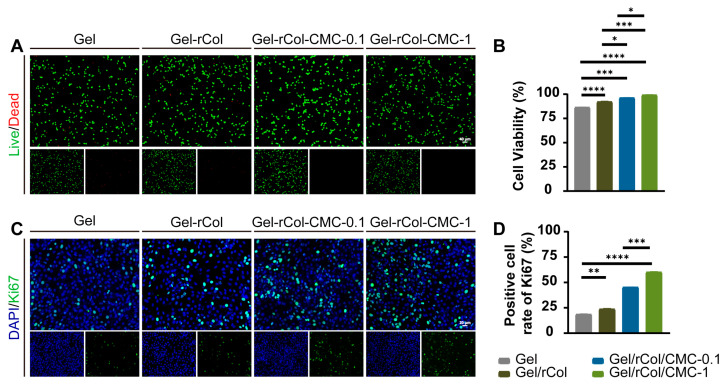
Cytocompatibility and proliferative activity of Gel/rCol/CMC composite hydrogels in vitro. (**A**) Live/dead staining images of cells cultured on Gel, Gel/rCol, and Gel/rCol/CMC hydrogels with different CMC contents, showing viable (green) and dead (red) cells. (**B**) Quantitative analysis of cell viability based on live/dead staining. (**C**) Immunofluorescence staining of Ki67 (green) and nuclei (DAPI, blue) to evaluate cellular proliferative activity on different hydrogels. (**D**) Quantification of Ki67-positive cells. Scale bars: 40 μm (**A**) and 20 μm (**C**). Data are presented as mean ± SD, and statistical significance is indicated (* *p* < 0.05, ** *p* < 0.01, *** *p* < 0.001, **** *p* < 0.0001).

The cytocompatibility of the gelatin-based composite hydrogels was first evaluated using NIH 3T3 fibroblasts by a CCK-8 assay at 24 and 48 h (Figure S2). All hydrogel formulations exhibited good cell viability at both time points, indicating the absence of obvious cytotoxic effects following EDC/NHS-mediated crosslinking. The appropriate polysaccharide incorporation does not impair short-term cellular metabolic activity. Live/dead staining was further performed to visually assess cell survival after being co-cultured with the hydrogels for 24 h (Figure 3A). Quantitative analysis of cell viability further supported these observations (Figure 3B), showing significantly higher viable cell percentages in the composite hydrogels containing CMC, particularly in the Gel/rCol/CMC-1 group. This trend is consistent with the CCK-8 results and indicates that controlled CMC incorporation does not compromise cell survival. Cell proliferation was subsequently evaluated by Ki67 immunofluorescence staining (Figure 3C). The number of Ki67-positive cells was markedly increased in the composite hydrogels compared with Gel group, with Gel/rCol/CMC-1 exhibiting the highest proportion of proliferating cells (Figure 3D). While Gel/rCol/CMC-0.1 also enhanced proliferation relative to Gel and Gel/rCol, the effect was less pronounced than that observed for Gel/rCol/CMC-1. Taken together, these results demonstrate that the incorporation of rCol and an appropriate amount of CMC not only preserves cytocompatibility but also actively supports 3T3 fibroblast proliferation. The enhanced cellular responses observed in Gel/rCol/CMC-1 likely reflect a balanced combination of biochemical cues and matrix properties, providing a favorable environment for cell growth without the potential limitations associated with excessive polysaccharide content.

### 2.4. Effects of Composite Hydrogels on Cell Migration and Macrophage Phenotype In Vitro

**Figure 4:**
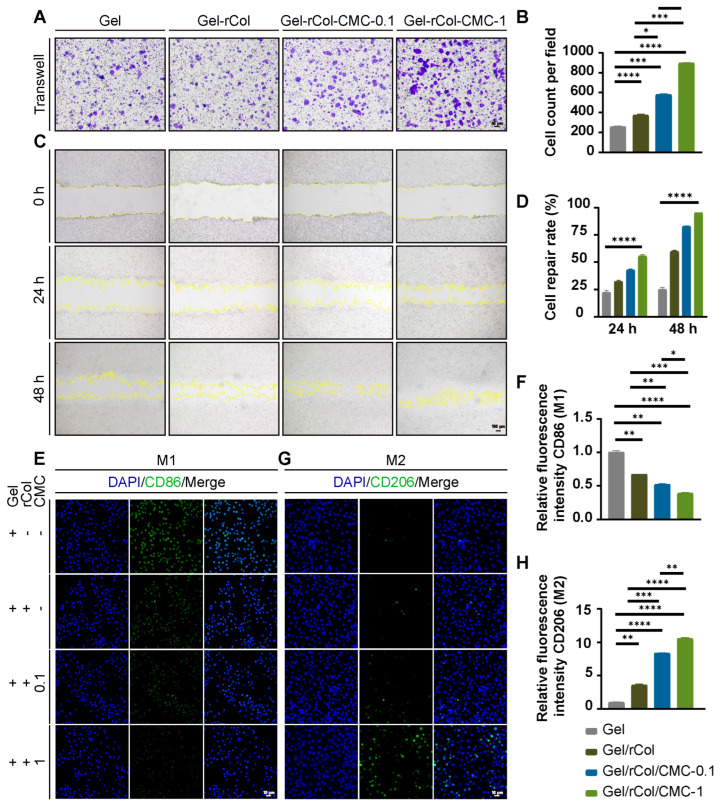
Effects of the composite hydrogels on cell migration and macrophage polarization in vitro. (**A**) Representative images of Transwell migration assays for cells cultured with Gel, Gel/rCol, and Gel/rCol/CMC hydrogels. (**B**) Quantification of migrated cells per field. (**C**) Representative images of scratch wound healing assays at 0, 24, and 48 h. (**D**) Quantitative analysis of cell repair rate. (**E**) Immunofluorescence staining of macrophage M1 marker CD86 (green) and nuclei (DAPI, blue). (**F**) Quantification of relative fluorescence intensity of CD86. (**G**) Immunofluorescence staining of macrophage M2 marker CD206 (green) and nuclei (DAPI, blue). (**H**) Quantification of relative fluorescence intensity of CD206. Scale bars: 40 μm (**A**), 100 μm (**C**), and 10 μm (**E**,**G**). Data are presented as mean ± SD, and statistical significance is indicated (* *p* < 0.05, ** *p* < 0.01, *** *p* < 0.001, **** *p* < 0.0001).

The effects of the composite hydrogels on cell migratory behavior were first evaluated using Transwell and scratch wound healing assays (Figure 4A,C). Compared with the Gel group, the incorporation of rColIII significantly enhanced cell migration, as evidenced by increased numbers of migrated cells and accelerated wound closure. Notably, Gel/rCol/CMC hydrogels further promoted migratory activity in a formulation-dependent manner, with Gel/rCol/CMC-1 exhibiting the highest cell migration and repair rates among the tested groups. Quantitative analysis confirmed a significant increase in migrated cell counts and scratch closure rates for Gel/rCol/CMC groups compared with Gel and Gel/rCol hydrogels (Figure 4B,D).

Macrophage polarization was further examined to assess the immunoregulatory potential of the composite hydrogels. Immunofluorescence staining revealed that macrophages cultured with Gel/rCol/CMC hydrogels displayed reduced expression of the M1-associated marker CD86 and increased expression of the M2-associated marker CD206 compared with Gel and Gel/rCol groups (Figure 4E). Quantitative analysis demonstrated a formulation-dependent decrease in CD86 fluorescence intensity and a corresponding increase in CD206 expression, with Gel/rCol/CMC-1 showing the most pronounced shift toward an M2-associated phenotype (Figure 4F,H). These results indicate a tendency toward a pro-regenerative macrophage phenotype. Together, the enhanced cell migration and favorable macrophage-associated responses suggest that rational incorporation of CMC into Gel/rCol matrices creates a microenvironment that supports key cellular functions relevant to wound repair, providing a functional basis for subsequent in vivo wound healing evaluation. Based on these findings, Gel/rCol/CMC-1 was identified as the optimal formulation due to its balanced mechanical integrity, swelling characteristics, and pro-regenerative cellular responses, whereas Gel/rCol/CMC-0.1 served as a lower-CMC reference to assess whether sufficient CMC incorporation was required to achieve functional benefits. These two formulations were therefore selected for in vivo wound healing experiments.

### 2.5. Effects of Composite Hydrogels on Full-Thickness Wound Closure In Vivo

**Figure 5:**
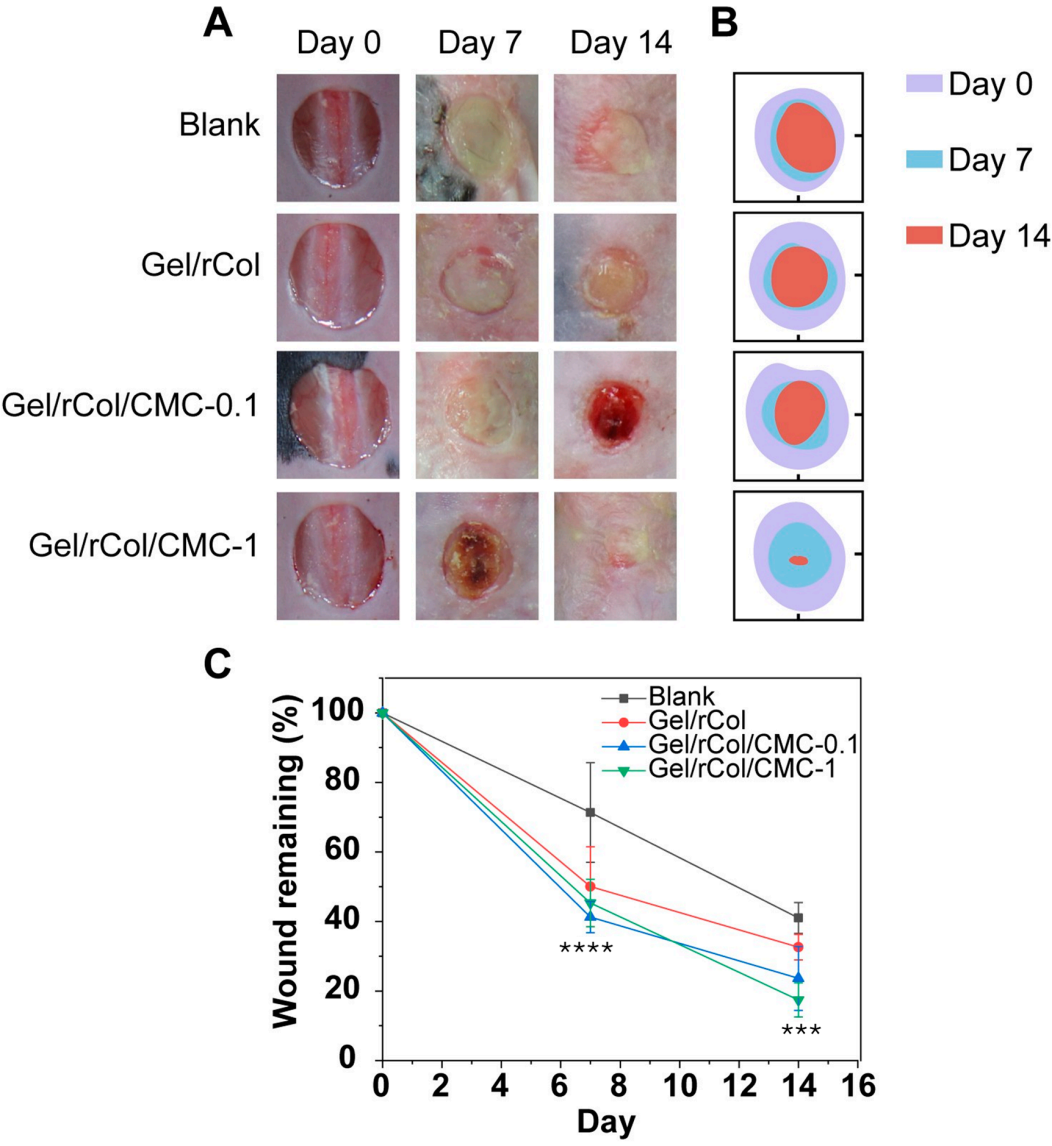
In vivo wound healing performance of Gel/rCol/CMC composite hydrogels in a full-thickness diabetic skin wound model. (**A**) Representative macroscopic images of wounds treated with different groups at days 0, 7, and 14 post-wounding. (**B**) Schematic representation of wound area changes over time. (**C**) Quantitative analysis of wound remaining area during the healing process. Data are presented as mean ± SD, and statistical significance is indicated (*** *p* < 0.001, **** *p* < 0.0001).

The in vivo wound healing performance of the composite hydrogels was further evaluated using a full-thickness skin defect model in streptozotocin (STZ)-induced diabetic mice (Figure 5). Representative macroscopic images of wound closure at days 0, 7, and 14 are presented in Figure 5A. Wound appearance and area were recorded at predetermined time points throughout the observation period. Compared with the other treatment groups, wounds treated with Gel/rCol/CMC-1 exhibited a markedly improved macroscopic healing profile. At day 7, the Gel/rCol/CMC-1 group showed a cleaner wound surface with reduced exudation and more granulation tissue formation, whereas wounds in the other groups remained partially covered with exudate and displayed delayed closure. By day 14, Gel/rCol/CMC-1 treated wounds were nearly completely closed, with only minimal residual defect area, while incomplete healing was still observed in the other groups. Quantitative analysis of wound area reduction further confirmed these observations (Figure 5B,C). At the end of the 14-day period, the wound area in the Gel/rCol group was slightly smaller than that in the Blank group, indicating a modest improvement in healing. The Gel/rCol/CMC-0.1 group exhibited a more pronounced reduction in wound area, whereas the Gel/rCol/CMC-1 group demonstrated the fastest wound closure, with significantly less remaining wound area compared with all other groups. Collectively, these results indicate that incorporation of an appropriate amount of CMC into the Gel/rCol hydrogel markedly enhanced wound healing efficiency in diabetic mice. Consistent with their compositions (Table S1), the protein-only Gel/rCol scaffold (gelatin + rCol) provided limited improvement over the Blank group, whereas the progressive incorporation of CMC in Gel/rCol/CMC-0.1 and Gel/rCol/CMC-1, which increases the polysaccharide content and network density, resulted in stepwise enhancement of wound closure, with Gel/rCol/CMC-1 showing the greatest effect.

### 2.6. Histological Evaluation of Tissue Regeneration and Extracellular Matrix Remodeling

**Figure 6:**
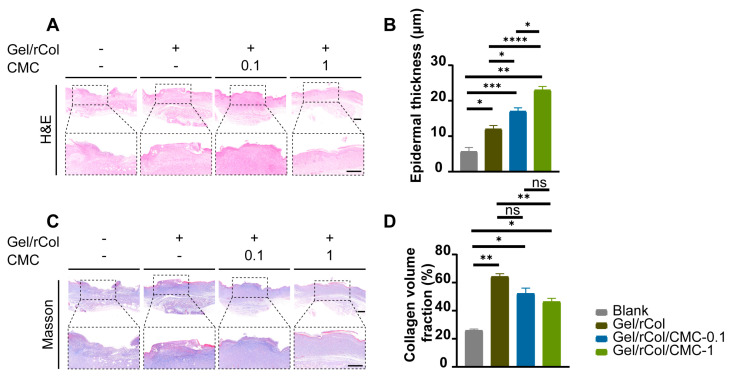
Histological evaluation of wound healing following treatment with composite hydrogels. (**A**) Representative H&E staining images of wound tissues collected at day 14 post-treatment. (**B**) Quantitative analysis of epidermal thickness based on H&E staining. (**C**) Representative Masson’s trichrome staining images illustrating collagen deposition and tissue remodeling in wound sites. (**D**) Quantification of collagen deposition from Masson’s trichrome staining. Scale bars: 1 mm for (**A**) and (**C**). Data are presented as mean ± SD, and statistical significance is indicated (ns, not significant, * *p* < 0.05, ** *p* < 0.01, *** *p* < 0.001, **** *p* < 0.0001).

Histological analyses were performed to further assess the quality of tissue regeneration following different treatment. Hematoxylin and eosin (H&E) staining revealed clear differences in epidermal regeneration and tissue architecture among the groups (Figure 6A). The Blank group exhibited incomplete epidermal coverage and non-migrative keratinocyte around the wound edge, indicating delayed re-epithelialization. In contrast, treatment with Gel/rCol promoted more continuous epidermal formation, while the incorporation of CMC further improved epidermal regeneration. Notably, the Gel/rCol/CMC-1 group displayed a well-formed, stratified epidermis with more uniform thickness. Quantitative analysis confirmed these observations, showing a progressive increase in epidermal thickness from the Blank and Gel/rCol groups to the CMC-containing composite hydrogels (Figure 6B). Among all groups, Gel/rCol/CMC-1 exhibited the greatest epidermal thickness, indicating enhanced re-epithelialization during wound healing.

Masson’s trichrome staining was used to evaluate collagen deposition and remodeling within the regenerated dermis (Figure 6C). The Blank group showed loose and disorganized collagen fibers, whereas Gel/rCol treatment resulted in increased collagen deposition. The Gel/rCol/CMC hydrogels promoted collagen accumulation with improved organization. Quantitative assessment showed significantly higher collagen deposition areas in the Gel/rCol/CMC groups, with Gel/rCol/CMC-1 achieving the highest level of collagen accumulation (Figure 6D). In conclusion, the histological evaluation results of H&E and Masson staining showed that Gel/rCol/CMC-1 treatment significantly promoted the process of wound regeneration and remodeling. These histological trends parallel the scaffold compositions, as increasing CMC content yields a more hydrated and structurally reinforced matrix that supports re-epithelialization and collagen remodeling.

### 2.7. In Vivo Angiogenic Response During Wound Healing

**Figure 7:**
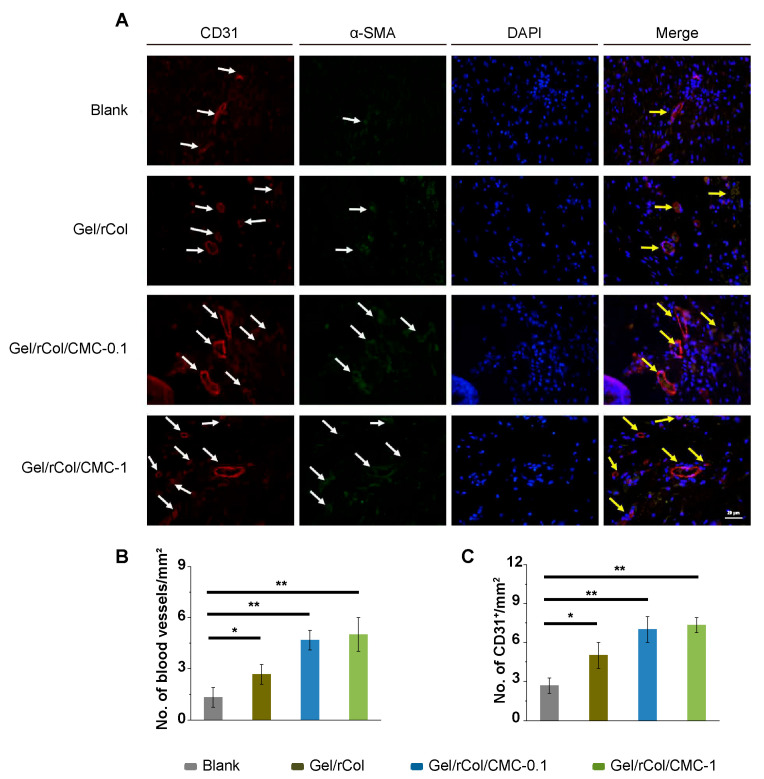
Angiogenesis and vascular maturation in wound tissues following different composite hydrogel treatments. (**A**) Representative immunofluorescence images of wound sections stained for CD31 (endothelial marker, red), α-SMA (smooth muscle cell marker, green), and nuclei (DAPI, blue). Arrows indicate representative blood vessels. (**B**) Quantification of blood vessel density per mm^2^. (**C**) Quantification of CD31-positive vessels per mm^2^. Scale bar: 20 μm. Data are presented as mean ± SD, and statistical significance is indicated (* *p* < 0.05, ** *p* < 0.01).

Angiogenesis within the regenerated wound tissue was further evaluated by immunofluorescence staining for endothelial and vascular maturation markers (Figure 7). CD31 staining revealed sparse and discontinuous microvessels in the Blank group, whereas increased numbers of CD31-positive structures were observed in Gel/rCol-treated wounds (Figure 7A). In comparison, Gel/rCol/CMC-treated groups exhibited a markedly higher density of CD31-positive vessels and well-distributed vascular structures. Quantitative analysis confirmed a significant increase in both total blood vessel density and CD31-positive vessel numbers in Gel/rCol/CMC-treated wounds compared with the Blank and Gel/rCol groups (Figure 7B,C). These results indicate that incorporation of CMC into the composite hydrogel enhances angiogenic responses in vivo in a formulation-dependent manner. Importantly, both Gel/rCol/CMC formulations promoted angiogenesis. In addition to endothelial marker expression, α-SMA staining was used to assess vascular maturation. Gel/rCol/CMC-treated wounds displayed increased α-SMA-positive signals surrounding CD31-positive structures, suggesting the formation of more mature and stabilized blood vessels. The colocalization of CD31 and α-SMA signals observed in merged images further supports enhanced vascular maturation in these groups. Together, these in vivo findings demonstrate that the composite Gel/rCol/CMC hydrogels supports coordinated wound repair by promoting vascular formation and tissue regeneration.

To evaluate in vivo biocompatibility, subcutaneous implantation studies were performed. Macroscopic observation showed gradual degradation of the hydrogels without signs of infection or adverse tissue reactions (Figure S3). Histological examination of the samples at week 2 after scaffold implantation using HE and Masson’s trichrome staining further confirmed good tissue integration and the absence of severe inflammatory responses or fibrotic encapsulation (Figure S4). These results demonstrate that the composite hydrogels exhibited favorable in vivo biocompatibility and controlled degradability, supporting their suitability for wound repair and regeneration applications.

## 3. Conclusions

In this study, a series of gelatin-based composite hydrogels incorporating rColIII and CMC were developed and systematically evaluated for skin wound healing applications. By modulating the composition of rColIII and CMC, the mechanical properties and microstructure of the hydrogels could be effectively adjusted, allowing the identification of an optimized formulation with balanced structural stability and biological performance. The selected Gel/rCol/CMC-1 hydrogel exhibited favorable cytocompatibility and supported key cellular functions associated with wound repair, including cell migration and a regenerative macrophage phenotype in vitro. In vivo evaluation using a full-thickness skin defect model in diabetic mouse demonstrated accelerated wound closure, improved tissue organization, enhanced collagen remodeling, and increased vascular formation and maturation. Overall, this work highlights the potential of combining gelatin, recombinant type III collagen, and polysaccharide components to construct bioactive hydrogel dressings with tunable properties. The tunability of the gelatin/rColIII/CMC system offers a practical basis for further optimizing mechanical resilience, hydration, and degradation kinetics, and for extending evaluation to more complex or chronic wound models. This work highlights the potential of combining gelatin, recombinant collagen, and polysaccharide components to construct bioactive hydrogel dressings with tunable properties. The findings provide a practical basis for the further development of immunomodulatory and regenerative hydrogel systems for skin repair.

## 4. Materials and Methods

### 4.1. Materials

Recombinant collagen type III (rCol) was purchased from Jiangsu Chuangjian Medical Technology Co., Ltd., Jiangsu, China. Dulbecco’s. Gelatin (porcine, type B) and CMC were purchased from Sigma-Aldrich, Beijing, China. N-(3-dimethylaminopropyl)-N-ethylcarbodiimide hydrochloride (EDC) and N-hydroxysuccinimide (NHS) were purchased from Sigma-Aldrich. Collagenase type II was purchased from Worthington, USA. All other chemicals were analytical grade and used without further purification. Dulbecco’s Modified Eagle’s Medium (DMEM), fetal bovine serum (FBS), and penicillin/streptomycin were purchased from Invitrogen, Shanghai, China. CCK-8 solution and live/dead staining reagent were purchased from Beyotime, Shanghai, China. DAPI and Phalloidin were purchased from Solabio Science & Technology Co., Ltd., Beijing, China. Anti-Ki67 antibodies, anti-CD31 and α-SMA were purchased from Abcam, Cambridge, UK.

### 4.2. Preparation of Scaffolds with Different Formulations

Five types of scaffolds were fabricated using the EDC/NHS crosslinking method from previously reported work [34]. To create the Gel/rCol/CMC scaffolds, CMC was first dissolved in MES solution and then mixed with EDC and NHS solutions. After 30 min of stirring, gelatin solution was added to the mixed solution and stirred before rCol was added to the mixture. Hydrogel scaffolds with different rCol concentrations were prepared. Pure gelatin and Gel/rCol hydrogel scaffolds were prepared using the same method as control groups. Different hydrogel formulations were prepared by mixing gelatin, rCol III, and CMC at fixed mass ratios of 10:1:x, where x = 0, 0.1, 1, or 2. Accordingly, the compositions were denoted as Gel/rCol/CMC-0.1 (10:1:0.1), Gel/rCol/CMC-1 (10:1:1), and Gel/rCol/CMC-2 (10:1:2). EDC and NHS were added at an EDC/NHS molar ratio of 2:1 at a 2-fold molar excess relative to CMC carboxyl groups for all Gel/rCol/CMC formulations. A Gel/rCol formulation (10:1) and a gelatin alone (Gel) formulation were also prepared. The detailed compositions of all five scaffolds are summarized in Table S1. The formed hydrogels were immersed in DI water to exclude the unreacted agents.

### 4.3. Rheological Analysis of the Hydrogel Scaffolds

Rheological analysis was performed to determine the mechanical properties of the scaffolds using a TA rheometer (HR-2, Shanghai, China) equipped with an 8 mm parallel plate. The hydrogel scaffolds (200 μL each sample) prepared as mentioned above were added to the HR-2 plate. The upper plate was lowered to a gap size of 1 mm, and samples were tested. The storage modulus (G′) and loss modulus (G″) were recorded at 25 °C under a frequency of 1.0 Hz and a strain of 1%.

### 4.4. Swelling and Enzymatic Degradation of Hydrogel Scaffolds

The swelling behavior was measured by monitoring the change in mass of dry and wet scaffolds. Freeze-dried scaffolds were used for the swelling and enzymatic degradation experiments. The hydrogel samples were placed in containers and pre-frozen in a −80 °C freezer for 24 h to ensure complete solidification. Subsequently, the pre-frozen samples are promptly transferred to the pre-cooled sample chamber (typically below −50 °C) of a lyophilizer vacuum freeze dryer (SCIENTZ-10N/A, Ningbo, China) and subjected to lyophilization for approximately 3 days. Freeze-dried scaffolds were first weighed and fully immersed in PBS buffer. The scaffolds were wiped with filter paper to remove extra buffer before weighing the wet mass. The swelling ratio was calculated as the ratio of swollen gel weight to dry protein weight.

The enzymatic degradation of hydrogel scaffolds was investigated by immersing 10 mg of each matrix in D-PBS buffer containing Collagenase II. The in vitro degradation rate was determined by measuring the remaining amount (Wt) of the matrix after incubation with 0.5 U/mL Collagenase II for a specified time period, until the matrix was completely degraded. To ensure accurate measurements, the microtubes containing the samples were inverted and checked for any excessive media along the inner walls of the tube for at least 1 min before weighing. The same amount of fresh ultrapure water was added to the tubes after each weighing.

The relative remaining mass of the scaffold during enzymatic degradation was calculated as follows:

(Wt/W0) × 100%,

where W0 is the initial dry weight of the scaffold, and Wt is the remaining dry weight at each time point.

### 4.5. Morphology of the Scaffolds

The morphology and porosity of the hydrogel scaffolds were characterized by scanning electron microscopy (SEM). Prior to SEM imaging, the hydrogel samples were frozen at −80 °C, followed by freeze-drying (0.05 mbar, −50 °C, 24 h), and sputter-coated with gold. The scaffolds were cut in half using a razor blade and mounted on an aluminum stub. The samples were coated with a thin layer of gold before observation using an AMETEK^®^ Quanta 3D FEG machine (Berwyn, IL, USA). Quantitative analysis of the pore microstructure of the SEM image was carried out by Image J software (Version 1.54p).

### 4.6. In Vitro Biocompatibility and Proliferation

*CCK-8 assay:* The in vitro cytotoxicity of the extract of the scaffolds was tested by a standard CCK-8 assay using 3T3 cells according to the instructions of ISO 10993. First, the hydrogel extract was prepared by immersing 1 g of hydrogel scaffold in 5 mL of full DMEM cell culture media at 37 °C for 24 h. In the meantime, 3T3 cells were seeded in a 96-well plate with a concentration of 8000 per well and left to attach overnight. The hydrogel extract was diluted to a series of concentrations (0.625, 1.25, 2.5, 10 mg/mL). The hydrogel extracts were added to the wells and co-cultured with 3T3 cells for 24 h and 48 h at 37 °C. Full cell culture with no extract was used as the control. Subsequently, 100 μL of fresh DMEM and 10 μL of CCK-8 solution (Beyotime, Shanghai, China) were added to each well for a 1 h of incubation at 37 °C, and the absorbance at 450 nm was measured using a microplate reader. The viability was calculated as follows.

Viability (%) = 100 × (OD450 nm of extraction)/(OD450 nm of control).

*Live/Dead staining*: The 3T3 cells were co-cultured with the hydrogel extracts at a concentration of 1 × 10^5^/mL, respectively. Live/dead staining was performed after 24 h of culture at 37 °C following the instructions of the manufacture. Images were taken by an inverted fluorescent microscope. The viability was calculated as follows.

Viability (%) = 100 × (Viable cells/Total cells).

*Ki67 immunohistochemistry:* Ki67 immunohistochemistry was used to evaluate the proliferation of 3T3 cells. Cells were fixed with 4% paraformaldehyde, permeabilized with 0.1% Triton X-100, stained with anti-Ki67 antibodies (1:200, Abcam, Cambridge, UK) at 4 °C for one night, and then incubated with AF488-conjugated secondary antibody (1:100, Abcam, Cambridge, UK) for 1 h at room temperature. 4′,6-diamidino-2-phenylindole (DAPI) was used to detect the nuclei in immunofluorescence staining. The number of Ki67-positive cells was counted using Image J software. The calculation formula for the positive cell ratio was as follows.

Positive cell rate of Ki67 (%) = 100 × (Number of positive cells/Total number of cells)

*Transwell assay*: We used 6-well Transwell inserts with 8 μm pore size filters and 6-well culture plates. Cells were suspended in medium containing 10% FBS and plated at 5 × 10^5^ cells per well in the upper chamber. Then, 1 mL of medium containing hydrogel extract was added to the lower chamber. After 24 h of incubation, cells on the upper surface of the filter membrane were removed with a cotton swab, and the migrated cells on the lower side of the filter membrane were stained with crystal violet. Photos were taken at every time point in each group. Positive cells were counted by Image J software.

*Cells wound scratch assay:* The proliferation of 3T3 cells was recorded using an FSX100 microscope. The proliferation distance of cells at every indication time was observed and analyzed as a ratio of proliferation area to original area. The cell scratch assay was performed using 3T3 cells. First, 1 mL of 3T3 cells (1 × 10^5^ cells/well) was seeded in a 12-well plate and placed in a 37 °C incubator with 5% CO_2_ for 24 h. A 200 μL pipette tip was used to induce a scratch in the 3T3 cells at a density of 70–80% per well. The 70–80% confluence allowed for both migration and proliferation over the 48 h of observation, avoiding contact inhibition of 3T3 cells at 90–100% confluence. Cell closures were recorded by microscope at each time point. The proliferation rate was determined as the ratio of proliferated area to original area. The fusion of cells was recorded by a microscope with an initial cell density of 1 × 10^5^, and photos were taken at every time point in each group. The percentage of wound closure was calculated for each field using the following formula.

Cell repair rate (%) =100 × [(Area at 0 h − Area at t h)/Area at 0 h].

*Phenotype Assessment of Macrophage Polarization*: For immunofluorescence, macrophages were seeded in confocal microscopy dishes for 24 h and subsequently activated with LPS (1 mg/mL) for 24 h. Macrophages were incubated with different hydrogels for another 24 h before fixing with 4% paraformaldehyde. Then, macrophages were incubated with primary antibodies against CD86, and CD206 at 4 °C for 12 h, and then incubated with respective fluorescent-labeled secondary antibodies for 1 h. Cell nuclei were stained with DAPI. Finally, samples were observed by confocal microscopy, and the fluorescence intensity was calculated with ImageJ software (Version 1.54p). The calculation formula for the positive cell ratio was as follows:

Positive cell rate (%) = 100 × (Number of positive cells/Total number of cells)

### 4.7. Animal Experiments

Male C57BL/6 mice weighing 20–25 g were utilized to establish STZ-induced diabetic wounds and to create an excisional skin wound model. Sprague–Dawley (SD) male rats 8–10 weeks in age with body weights ranging from 220 to 260 g were used for subcutaneous hydrogel implantation for biodegradation and biosafety tests. The animals were fed ad libitum water and a rodent diet. All procedures were approved by the Laboratory Animal Resources Center of Wenzhou Medical College (China), Approval No. xmsq2023-0462.

### 4.8. In Vivo Degradation and Tissue Integration of the Hydrogel Scaffolds

SD rats were used to test the in vivo degradation and tissue integration of the hydrogel scaffolds by subcutaneous implantation. A total of 36 SD rats were randomly assigned to three groups (Gel/rCol, Gel/rCol/CMC-0.1, and Gel/rCol/CMC-1), with *n* = 12 animals per group.

Rats were anesthetized with intraperitoneal injection of 10% chloral hydrate (3.0 mg/kg). Two subcutaneous pockets were made on the dorsum of each animal by blunt dissection, with two on each side. Each hydrogel weighing 200 mg was implanted in each pocket, and the incision was sutured with silk stitches. Implants were sampled every week until no obvious hydrogel remained (five samples for each time point). Gross images were recorded to observe the remaining hydrogels and the potential abnormal response.

### 4.9. In Vivo Wound Healing Tests

The wound healing effects of the scaffolds were tested in STZ-induced diabetic C57BL/6 mice. A total of 48 STZ-induced diabetic mice were randomly assigned into four groups (Blank, Gel/rCol, Gel/rCol/CMC-0.1, and Gel/rCol/CMC-1), with *n* = 12 animals per groups.

One wound on the dorsal surface of each mouse were created using a sterile 1 cm punch biopsy tool. Full-thickness wounds extending through the panniculus carnosus were excised using a sterile scissor. The treatment was then applied to the wounds. There were three treatment groups, including a saline control. The wounds were covered with sterile gauze after treatment. Digital photographs of the wounds were taken on the day of surgery and every 3 days thereafter. Wound closure rate was determined by measuring the wound area and calculated as the percentage of the original wound.

Hematoxylin and eosin (H&E) and Masson’s trichrome were used to visualize the in vivo degradation, tissue integration, and neo-vessel formation of the samples following the manufacturer’s instruction for use. After staining, the prepared slides were examined under an optical microscope to gain insight into the structure and composition of the tissue.

Epidermal thickness was measured on H&E-stained vertical skin sections. For each sample, five random fields were selected. In each field, three perpendicular measurements from the basal layer to the stratum corneum were taken using Image J software. The mean epidermal thickness for each sample was calculated by averaging all individual measurements. Group means were then derived from multiple biological replicates.

Quantification of collagen deposition: Collagen deposition was assessed in Masson’s trichrome-stained sections. Five randomly selected fields per sample were captured under high magnification. Using ImageJ software with the color deconvolution plugin, the blue-stained collagen fibers were specifically isolated and thresholded. The collagen volume fraction (CVF) was calculated as follows.

CVF (%) = (Area of blue staining/Total tissue area) × 100.

The mean CVF from all fields was used for statistical analysis.

CD31 and α-SMA immunohistochemistry: The sections were fixed in acetone at −20 °C for immunofluorescence treatment. The sections were stained with CD31 and α-SMA antibodies. After washing with PBS, the sections were incubated with secondary antibody. For double immunofluorescence staining of CD31 and α-SMA, paraffin sections were first dewaxed and washed with PBS. Then, the sections were blocked with serum and incubated with anti-CD31 primary antibody and anti-α-SMA antibody. Finally, the sections were stained with DAPI and photographed. Image J was used to count the number of CD31^+^-stained vessels and the number of mature vessels stained with both CD31 and α-SMA.

### 4.10. Statistical Analysis

All values are expressed as mean ± standard deviation (SD). Statistical analysis was performed using GraphPad Prism (Version 8.0.2), Origin (Version 2025b), and Image J software (Version 1.54p). We analyzed the comparisons between groups using Student’s *t* test, one-way ANOVA followed by Tukey’s multiple comparisons test, and Dunnett’s multiple comparisons test. Differences were considered statistically significant when ns, not significant, * *p* < 0.05, ** *p* < 0.01, *** *p* < 0.001, and **** *p* < 0.0001.

## Data Availability

The data presented in this study are available on request from the corresponding authors.

## Supplementary Materials

The following supporting information can be downloaded at https://www.mdpi.com/article/10.3390/gels12020142/s1, Table S1, Figures S1–S4, and supporting Materials and Methods.
