# Supplementary material for: Design and Biological Evaluation of a Gelatin/Recombinant Type III Collagen/CMC Composite Hydrogel for Wound Healing
Source: Gels. 2026 Feb 3;12(2):142. doi: 10.3390/gels12020142 (PMC12940753; doi:10.3390/gels12020142)
Supplement: Supplementary file 1 [file gels-12-00142-s001.zip › gels-4096132-supplementary.pdf]

Supporting Information

Supporting Figure S1-Figure S4

Supporting Table 1

Table S1 Compositions of the five hydrogel formulations.

Sample	Gel (wt%)	rCol (wt%)	CMC (wt%)
Gel	1.58	0	0
Gel/rCol	1.58	0.158	0
Gel/rCol/CMC-0.1	1.58	0.158	0.0158
Gel/rCol/CMC-1	1.58	0.158	0.158
Gel/rCol/CMC-2	1.58	0.158	0.316

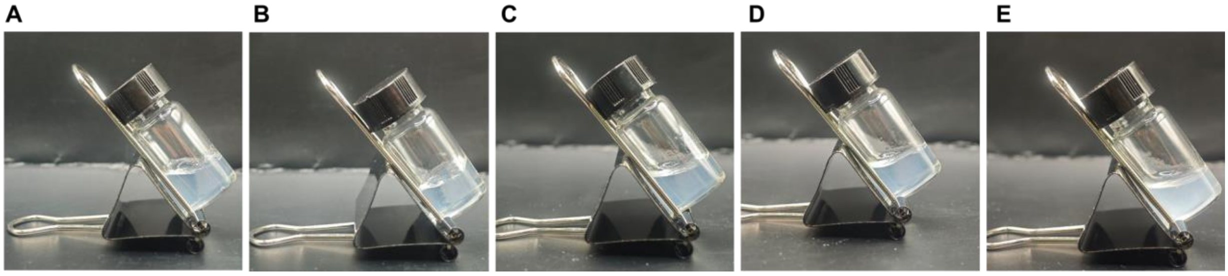

Figure S1. Representative images of the hydrogel scaffolds obtained from each formulation.

A) Gel hydrogel.

B) Gel/rCol hydrogel.

C) Gel/rCol/CMC-0.1 hydrogel.

D) Gel/rCol/CMC-1 hydroge.

E) Gel/rCol/CMC-2 hydrogel.

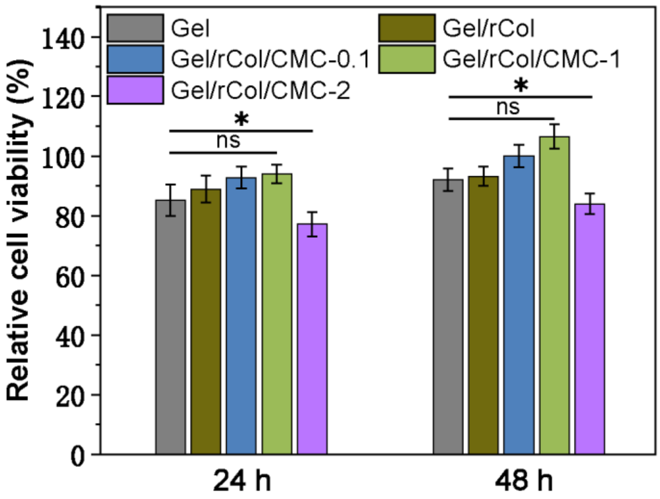

Figure S2. *In vitro* cytocompatibility evaluation of Gel/rCol/CMC composite hydrogels by CCK-8 assay. Relative cell viability of cells cultured with Gel, Gel/rCol, and Gel/rCol/CMC hydrogels with different CMC contents (0.1, 1, and 2) after 24 h and 48 h incubation, as determined by CCK-8 assay. Data are presented as mean ± SD. Statistical significance is indicated (ns, not significant, *$p < 0.05$)

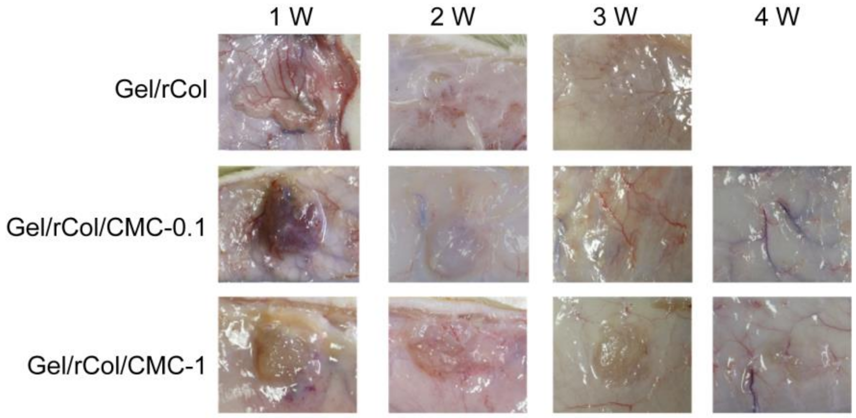

Figure S3. *In vivo* hydrogel degradation and tissue integration. Gross images of implanted hydrogels and surrounding tissues. The Gel/rCol material was not observed by week 4 after implantation.

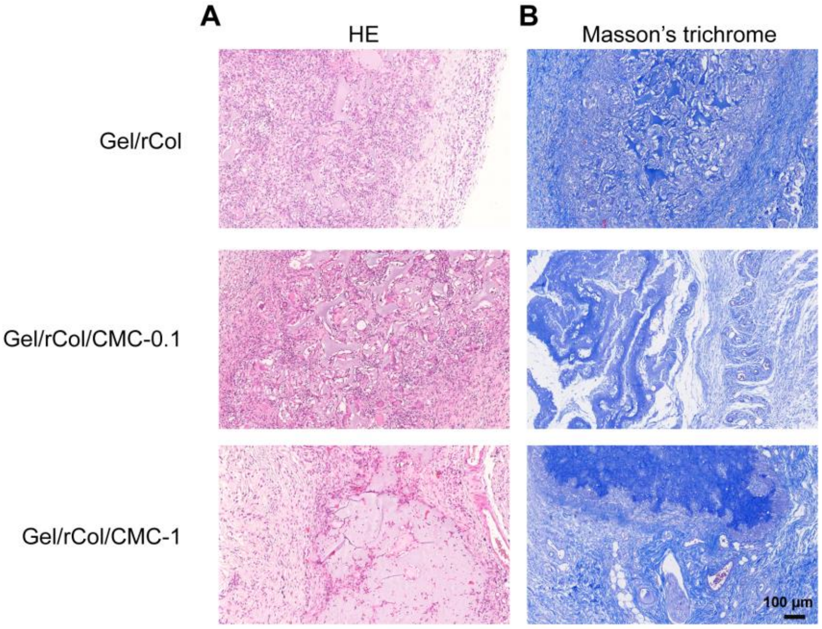

Figure S4. *In vivo* subcutaneous implantation of the hydrogels. HE (A) and Masson's trichrome (B) staining of samples at week 2. Scale bar: 100 μm.
